# Gradient Material Strategies for Hydrogel Optimization in Tissue Engineering Applications

**DOI:** 10.3390/ht7010001

**Published:** 2018-01-04

**Authors:** Laura A. Smith Callahan

**Affiliations:** The Vivian L. Smith Department of Neurosurgery, Center for Stem Cell & Regenerative Medicine, and Department of Nanomedicine and Biomedical Engineering, McGovern Medical School, University of Texas Health Science Center at Houston, Houston, TX 77030, USA; laura.a.smithcallahan@uth.tmc.edu; Tel.: +1-713-500-3431

**Keywords:** gradient, combinatorial method, cell–material interface

## Abstract

Although a number of combinatorial/high-throughput approaches have been developed for biomaterial hydrogel optimization, a gradient sample approach is particularly well suited to identify hydrogel property thresholds that alter cellular behavior in response to interacting with the hydrogel due to reduced variation in material preparation and the ability to screen biological response over a range instead of discrete samples each containing only one condition. This review highlights recent work on cell–hydrogel interactions using a gradient material sample approach. Fabrication strategies for composition, material and mechanical property, and bioactive signaling gradient hydrogels that can be used to examine cell–hydrogel interactions will be discussed. The effects of gradients in hydrogel samples on cellular adhesion, migration, proliferation, and differentiation will then be examined, providing an assessment of the current state of the field and the potential of wider use of the gradient sample approach to accelerate our understanding of matrices on cellular behavior.

## 1. Introduction

To expedite progress toward clinical deployment of tissue engineering applications, biomaterial optimization is moving from traditional ad hoc approaches to combinatorial/high-throughput methods [1]. These methods have long been used to streamline pharmaceutical development, but have only recently been adapted by the biomaterials community [2]. A number of methods have been developed by the biomaterials community for matrix optimization. The three most common approaches are: design of experiments, a statistical approach that uses predictive modeling to determine a combination of experiments needed to achieve a desired biological outcome; arrays, which use a number of discrete samples to optimize biological response; and gradients, which alter sample composition down the length of the sample to provide information over a range of conditions to optimize the biological effect. Each of these approaches has its own advantages and drawbacks, which makes each particularly advantageous in certain circumstances [3]. A number of reviews exist on each of these methods and the general advantages of high-throughput approaches for matrix optimization [4,5,6]. This review will focus on the use of gradient material samples. Types of gradient samples constructed for biological studies and the observed changes in cellular response in gradient material samples will be covered.

**Figure 1:**
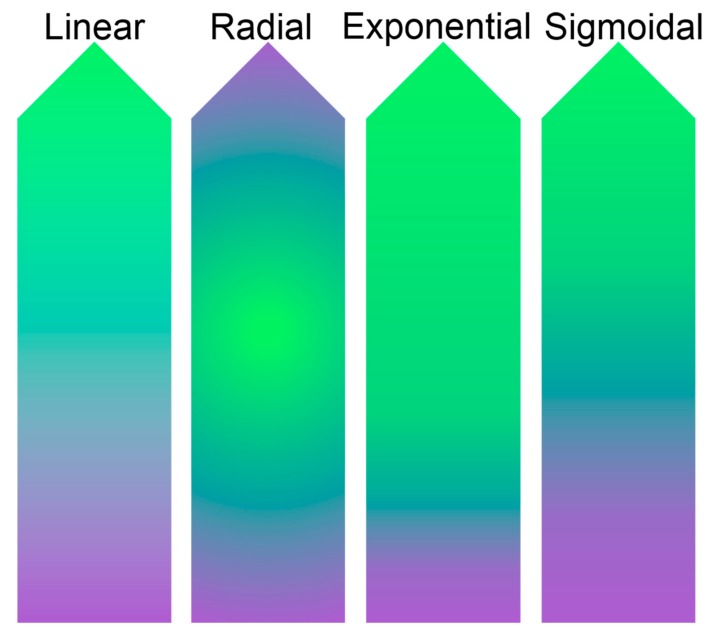
Schematic of gradient profiles in material samples.

The hallmark of the gradient approach is a gradual change in composition across the sample, but this change does not need to be linear as radial, exponential, and sigmoidal gradients (Figure 1) have been made [7,8,9]. Gradient material samples can be fabricated through a number of approaches, but the most common involve pumps filling molds using inverse pumping profiles or diffusion across a gap [10,11,12]. Rapid advances in technology development have led to less reliance on pumps to form the gradient using changes in light exposure to covalently bond [13,14] or cleave [15] matrix elements, surface tension [16], and capillary action [17] instead for gradient formation. To reduce the need for specialized equipment and promote greater access to gradient samples, layer deposition [18] and cooling from a localized source at one end of the mold [19] have been used to form gradient samples.

A major advantage of using a gradient material sample approach over design of experiments and arrays to understand cell–material interactions and optimize matrix conditions for biological response is the reduced variation in sample preparation between conditions in the test range because they are housed in a single sample. This reduced variation enables greater resolution of regions of cellular transitions, allowing for more accurate identification of thresholds in the test range. Increases in our understanding of complex biological interactions with biomaterials has led to the development of technologies to allow the fabrication of orthogonal gradients [14,20,21,22,23] in order to simultaneously study the effects of changes in multiple material properties on cellular behavior and examine interactions.

## 2. Types of Gradients Developed for Optimization of Biological Response to Materials

The high water content of hydrogels, which is similar to biological tissue, and their tailorability which enables them to meet the structural support and bioactive signaling needs for a number of cell types, have made hydrogels a widely used material platform in tissue engineering development [24]. Although other matrix platforms, like macroporous foam scaffolds, can also be fabricated as gradient samples [25,26,27], the review focuses on hydrogel gradient systems because hydrogel system support greater cell–material interaction for a longer period of time than macroporous scaffolds. As a number of material properties are known to affect cellular behavior, a number of gradient hydrogel systems, which will be discussed below, have been developed to tune cellular response. It is, however, important to note that the aqueous nature of hydrogels makes their material properties particularly susceptible to changes in the local environment (bioactive signaling interactions, cells, ions, etc.) compared to other tissue engineering matrix types, such as foams and nanofibers. These changes can complicate analysis of the cellular response because multiple material properties can change at once, but through characterization of the matrices can improve analysis of cellular response.

### 2.1. Composition

Traditionally, gradient approaches to these studies blend two different polymers [28,29,30,31], different molecular weights of the same polymer [32,33,34], or vary the mass fraction of the same polymer or monomer in the same hydrogel [10,35,36,37] in order to optimize the blending ratio. However, conversion of the reactive moieties during polymerization has also been examined [35,38,39]. This is important because reduced conversion is associated with reduced cellular viability [38,39], which makes identification of cellular thresholds for survival with a gradient approach particularly useful. One study employed orthogonal gradients of triethylene glycol dimethacrylate (TEGDMA) and 2,2-bis[*p*-2′-hydroxy-3′-methacryloxypropoxy]-phenyl]propane (BisGMA) concentration to identify that higher TEGDMA contents increased methacrylate conversion, while higher BisGMA content increased elastic modulus [39]. Another study, which blended hyperbranched macromers into polyethylene glycol (PEG) hydrogels identified that the resulting changes in crosslinking altered matrix stiffness, hydrophobicity, and surface roughness [30]. Changing of matrix composition is fundamentally one of the simplest and most important material properties that affect cellular response as numerous changes beyond changing chemistry available to the cells typically occur in the matrix (topography, porosity, wettability, stiffness, protein absorption, etc.) [30,40]. Although typically not the primary focus of these studies, the additional material and mechanical property changes need to be adequately characterized in order to fully understand the matrices effects on cellular behavior.

### 2.2. Material and Mechanical Properties

Many times changing a specific material or mechanical property in the matrix is the fabrication objective. Some of the most commonly examined material and mechanical properties using a gradient approach are topography, porosity, and stiffness. Techniques used to generate topography gradients in hydrogels range from changes in fiber density, thickness, composition, and oxidative wrinkling of the surface [28,30,41]. To generate porosity gradients, freeze-thawing techniques in composition gradients, and radical diffusion have been utilized [29,42,43]. One gradient porosity study found that by the addition of 0.1% acetic acid to the solvent system shifted the pore size range from 75–180 μm to 45–125 μm, providing a convenient way to tailor pore size without altering matrix composition [42]. Due to high interest in the effects of stiffness changes on cellular response (attachment, migration, differentiation, etc.) to biomaterials, a significant number of techniques have been developed to create stiffness gradients. Changes in polymer mass fraction [10,12,36,37], crosslinking agent [44,45], blending [34], hydrogel thickness [46], photomask [47], and lithography pattern [15] have all been employed to fabricate stiffness gradients. Overlapping gradients looking at the interplay of wettability and fibronectin concentration with changes stiffness have also been generated [14,48].

### 2.3. Bioactive Signaling

The extracellular environment of healthy tissue contains a number of bioactive signals, whose presence and concentration affect cellular behavior. To emulate these chemotactic signals or to optimize signaling to promote specific cellular behaviors, bioactive signaling molecule gradients in hydrogels have been formed. These bioactive signaling gradients typically have assumed one of two forms, tethered or released/freely diffusing gradients of the bioactive signaling molecule. The tethered format seeks to emulate some of the native bioactive signaling normally embedded in the matrix. To achieve this, a number of whole protein [49,50,51,52] and bioactive peptide [53,54,55,56,57]—which are short amino acid chains from proteins that bind cell receptors and influence behavior—gradients have been tethered in hydrogels. Strategies to achieve these tethered gradients have included gradient incorporation of the bioactive signaling element during fabrication [53,55,56,57], photomask micropatterning on the surface [51], and diffusion of the bioactive signaling molecule through the hydrogel where it covalently binds reactive moieties tethered to the polymer backbone [22,52], or on nanoparticles embedded in the matrix [58]. As bioactive signaling is complex, dual tethered gradients have been fabricated [49] and as our ability to characterize the materials and understanding of biological complexity increases, so likely will the number of overlapping gradients fabricated in a single sample.

Released bioactive signaling gradients seek to emulate chemotactic signals suspended in the extracellular milieu surrounding the matrix. A number of approaches have been developed for not only proteins [59,60,61,62,63], but also pharmaceuticals [20], short interfering RNA [64], and transcription factors [65]. Diffusion from a single source in or near the hydrogel [62], multiple micro/nanoparticles embedded in the hydrogel [59,60,66], gradient incorporation during fabrication [63], and capillary networks embedded within the hydrogels [17] have been used to create these types of bioactive signaling agent gradients. Like the tethered bioactive signaling of the extracellular matrix, these signaling cascades are complex. Nonlinear, inverse, and sequential gradients of multiple bioactive signaling agents have been formed to look at the effects of both concentration and timing in order to add to our understanding of natural tissue development [20,59,60,61,66,67].

### 2.4. Emerging Areas Where Gradient Studies Are of Potential Interest

It was not long ago that the entire extracellular matrix was thought to be inert [68], and that changes in the stiffness of the culture surface could affect differentiation were inconceivable [69]. Our understanding of environmental effects on cellular behavior is rapidly advancing and so are our topics of study. Emerging areas of study with gradients in matrices include oxygen concentration [70], intestinal flow [71], and matrix strain created by fluid flow [72]. Each may prove as transformative for the field as the extracellular matrix bioactivity and stiffness.

## 3. Understanding the Cell–Material Interface

Cellular interaction with the extracellular environment influences cellular behavior [73,74]. Through manipulation of the cellular–material interfaces, this cellular behavior can be guided toward desired outcomes, such as tissue formation or cytokine production [75]. Our understanding of how these complex interactions work together to alter cellular behavior is still limited. Therefore, a gradient approach to monitor the effects of fine changes in material properties on cellular outcomes is a powerful tool to increase our biological understanding. In this section, the observed changes in cellular attachment, migration, proliferation, and differentiation using gradient samples and the value of this type of approach to these studies to our biological understanding will be discussed.

### 3.1. Attachment

Most cell types require anchorage for survival, which means that changes in cellular attachment to a material is a first step toward influencing later cellular behavior (migration, proliferation, and differentiation) [76]. A number of material properties have been shown to affect cellular adhesion including composition [29,77], topography [41], wettability [48], stiffness [33,37,47,48,74], and bioactive signaling concentration [14,22,32,53,77]. The power of a gradient approach to refine material conditions to promote adhesion was demonstrated in a study that resolved a composition of at least 54.3% gelatin in a gelatin–chitosan composition gradient as necessary for elongation of smooth muscle cells, and that a composition of less than 10% gelatin resulted in cellular aggregate formation due to a failure of cells to spread [29]. This level of compositional refinement on cellular adhesion is often not detected in other combinatorial method approaches. Furthermore, a topographical study was able to identify an optimal feature size range of 0.4–2.6 μm amplitude and 4.0–7.1 μm wavelength to induce human mesenchymal stem cell (hMSC) alignment, due to alteration in focal adhesion number [41]. More advanced studies have examined the interplay between matrix stiffness and wettability or fibronectin concentration [48,74]. Both studies found an interplay between the second gradient condition and matrix stiffness that affected cellular spreading on the matrix, but changes in matrix wettability were found to significantly alter the matrix stiffness where maximal hMSC adhesion and spreading occurred [48].

Directly changing the adhesiveness of the materials through changing the concentration of bioactive adhesion moieties is the most straightforward way to study attachment to a matrix. This can be achieved using whole proteins [14,22] or peptides [32,53,56,77]. A number of studies have utilized the RGD (Arg-Gly-Asp) peptide from fibronectin, a highly studied adhesion peptide, in a gradient sample to examine cellular adhesion. Human umbilical vein endothelial cells (HUVEC) and fibroblasts studies have found that increasing RGD concentration increases cellular attachment and spreading [32,53,77]. However, it is important to note that a gradient surface study found high concentrations of RGD toxic to dendritic cells, which regulate immune response [78]. It is possible that the current RGD concentration gradient hydrogel adhesion studies do not present RGD concentration levels as high as the dendritic cell study did to the cells, that the different cell types used have different responses to RGD concentrations, or that additional material or mechanical property differences between studies are altering the results. Further investigation of these questions would be beneficial to improving our understanding of cellular adhesion of different cell types to similar materials.

### 3.2. Migration

Cells can sense steep gradients in stiffness and then tend to migrate up the stiffness gradient. This behavior is known as durotaxis. Studies using stiffness gradients have observed this behavior [9,23,44,46]. Several of these studies have noted that cells achieve higher speeds on softer matrices compared to stiffer ones [9,44]. Epithelial monolayer migration on a stiffness gradient has been shown to be organized by mechano-traction leading to polarization and directed migration, instead of the random walk that occurs on single stiffness matrices [46]. One study taking a more dynamic approach used a strain gradient to gradually stiffen a collagen matrix over the sample length, which led to greater invasion of mammary epithelial cells from organoids into the collagen matrix in stiffer regions [79]. Shallow stiffness gradients do not lead to durotaxis [80], and can be used to study cellular signaling transitions in cellular response to matrix stiffness changes, including durotaxis. Expression of lamin A, a mechanosensitive scaffold protein in the nuclear envelope, was differentially regulated by matrix stiffness in adipose stem cells and C2C12 myoblasts, indicating different mechano-sensitivities in the two cell types [18]. Further, mechanosensitive regulator Yes-associated protein (YAP) translocated to the nucleus over different stiffness ranges in the two cell types. Adipose stem cells nuclear translocation occurred between 12 and 20 kPa, while C2C12 myoblasts nuclear translocation occurred between 2 to 38 kPa [18]. These transition ranges had been difficult to identify with other methods.

Similar to durotaxis, chemotaxis is the movement of cells up a bioactive signaling gradient that can be either tethered to the matrix [22,31,53,66], or in the extracellular milieu [66,71]. A study of fibrosarcoma cells found that the cells change direction to continue to move toward increasing fetal bovine serum concentration when the direction of the concentration gradient is changed [66]. One study with fibroblasts found that once a concentration threshold was reached that fibroblast migration no longer increased with increasing fibronectin concentration [22]. Other studies have identified that different chemotactic responses in different cells types with some cell types responding to the gradient and migrating up it, while others maintain random walk patterns [31,54]. These studies underscore the need to examine cellular response for every cell type of interest in order to adequately design the material interface for desired cellular response.

### 3.3. Proliferation

Cellular expansion is necessary for the success of most tissue engineering applications. First, enough cells must be generated for the procedure, and then the starting population must expand to fill the matrix. Traditional cell culture conditions are not always optimal to identify be best conditions to achieve these design goals. Several gradient studies have used gradient approaches to identify the optimal matrix composition [29], stiffness [45,56], and bioactive signaling [51] or nutrient [17] concentrations for cellular proliferation. One study identified a threshold of 190 kPa stiffness in polyvinyl alcohol–hyaluronic acid matrices for maximal hMSC proliferation [45], but most selected the highest concentration tested as maximal concentration tested [17,29,51]. These later studies would benefit by expanding the tested range until a decline in proliferation is detected, as they may not have truly identified the optimal condition for cellular proliferation in their studies.

### 3.4. Differentiation

Since the fundamental work by Engler et al. [69] was published, there has been significant interest in effects of matrix stiffness on the differentiation of immature cells. Expanding on that original work, a recent study of hMSC cultured on a stiffness gradient in polyvinyl alcohol—hyaluronic acid matrices further refined the optimal stiffness ranges for different lineage selections to be ∼20 kPa for neurons, ∼40 kPa for myoblasts, ∼80 kPa for chondrocytes, and ∼190 kPa for osteoblasts [45] in that system. Another study using valvular interstitial cell found a stiffness of 32 kPa optimal for myofibroblast activation, with a minimum stiffness of 15 kPa necessary to initiate the transition [80]. In these studies, the use of a gradient approached facilitated the identification of these thresholds to a level that is difficult with other approaches.

**Figure 2:**
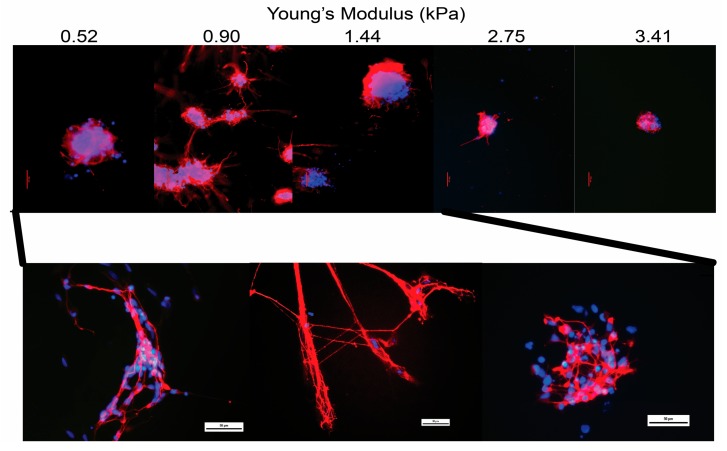
Staining of neural specific βIII tubulin (red) with nuclear stain (blue) in human induced pluripotent stem cell derive neural stem cells after 14 days of neural differentiation culture on polyethylene glycol hydrogels possessing a continuous gradient in Young's modulus. Scale bar is 100 µm across whole gradient and 50 µm enlargement. Modified and reprinted with permission of John Wiley & Sons, Inc. from [36].

Generally, immature cells, like hMSC, are thought to be more mechano-sensitive than the mature cell types generated from them. However, a recent study of human induced pluripotent stem cell (hiPSC)-derived neural stem cells (NSC) has indicated that at these derived progenitor cells may be more mechano-sensitive than previously thought [36]. Significant changes in axon network organization and messenger RNA (mRNA) gene expression were observed over ∼200 Pa change in matrix stiffness (Figure 2). Maximal axon extension and neural differentiation was observed at ∼900 Pa, which is significantly lower that the ∼20 kPa stiffness found in the previously discussed study [45]. A study of primary human chondrocytes found that reduced matrix stiffness (∼2 kPa) produced maximal glycosaminoglycan and collagen content in the matrix [12], which is significantly different than the ∼80 kPa that enhanced differentiation [45]. The studies utilize different processing techniques and polymers that likely contribute to these differences in observed results. Osteobastic differentiation has been more consistent. High-stiffness matrices have increased alkaline phosphatase and mineral content in both hMSC and progenitor cells [10,81]. These studies underline the complexity of matrix stiffness on cellular differentiation and the difficultly that occurs when comparing results between similar cell types. Even using a gradient approach, variations due to cellular original, differentiation state, and material platform lead to significant changes in optimal matrix stiffness.

Taking this examination of the complexity of matrix stiffness on cellular differentiation a step further, a study overlaid a fibronectin concentration gradient over the stiffness gradient. Human mesenchymal stem cells cultured in media containing both pro-osteogenic and adipogenic signals were cultured on the gradient. Both stiffness and fibronectin concentration were found to promote osteoblast differentiation, while only matrix stiffness affected adipogenic differentiation [14]. Another study examining the effect of RGD—a fibronectin-derived peptide—concentration on a single stiffness PEG matrix found the availability of crosstalk (secreted cytokines) from cells cultured in other test regions made the observed adipogenic differentiation RGD concentration dependent, but when crosstalk was eliminated, RGD concentration did not influence lineage choice of the hMSC [82]. In glioma cells, the expression of oncogenic microRNA miR18a was found to increase as both matrix stiffness and fibronectin concentration increased [14]. The further addition of cytokines secreted by macrophages led to high expression of miR18a regardless of matrix stiffness [14]. Although typically not considered, gradient samples can be constructed in either continuous gradient or gradient array formats to control access to crosstalk [83]. These studies demonstrate the ramification that the availability of crosstalk along the gradient can have on the results of biological experiments and the need to consider its effects during experimental design.

**Figure 3:**
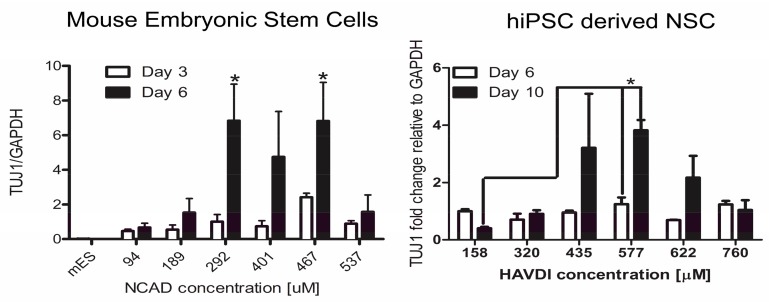
mRNA expression of βIII tubulin (TUJ1) in mouse embryonic stem cells and human induced pluripotent stem cell derive neural stem cells over a time course of neural differentiation on polyethylene glycol hydrogels possessing continuous concentration gradients of N-cadherin-derived peptide HAVDI (His-Ala-Val-Asp-Ile). GAPDH: Glyceraldehyde-3-phosphate dehydrogenase; NCAD: N-cadherin; hiPSC: human induced pluripotent stem cell; NSC: neural stem cells. Reprinted with permission from [85] with permission from Elsevier, Copyright 2016 Elsevier, Ltd., and [55], Copyright 2017 American Chemical Society.

To examine just the effects of bioactive signaling concentration on differentiation, a number of studies have used both released [65,66] and tethered [52,55,56,57,82,84,85] bioactive signaling approaches. Many of these studies have focused on the use of the gradient strategy to optimize neural differentiation. Using released orthogonal gradients, one study found that high concentrations of retinoic acid and smoothened agonist, a small molecule activator of the sonic hedgehog pathway, were necessary for motor neuron differentiation of embryonic stem cells [66]. Remaining neural studies have utilized tethered gradients of nerve growth factor [84], laminin [52], laminin-derived peptide IKVAV (Ile-Lys-Val-Ala-Val) [57], and n-cadherin-derived peptide HAVDI (His-Ala-Val-Asp-Ile) [55,85] to identify optimal concentrations for neurite extension and neural differentiation. In the IKVAV concentration study, a substantial shift from 570 µM to 60 µM in the optimal IKVAV concentration was noted for maximal neural differentiation when the cell culture was shifted from on the matrix surface to in the PEG hydrogel [57]. This indicates that re-optimization will likely need to occur as technology moves to 3D culture from 2D culture. Much like the biological complexity observed in the mechanical gradient studies, the range of optimal concentrations for maximal expression of neural differentiation markers in mouse embryonic stem cells and hiPSC derived NSC used in HAVDI studies was shifted to a higher concentration range for the hiPSC derived NSC (Figure 3) [55,85]. This data raises interesting issues as most biomaterial optimization is conducted with rodent cells. Given the rodent data using a traditional testing approach, there is significant potential that an HAVDI concentration below the threshold necessary to stimulate human response would be selected for the testing. With other bioactive signaling agents, the ranges between optimal signaling ranges may slide further apart, giving errant results in regards to species response to the signal, which generally requires more consideration in biomaterial development. However, employment of gradient approaches as a first optimization step with each cell type will mitigate the potential to miss these shifts in cellular response.

## 4. Future Directions and Conclusions

Appreciation for the complexity of cell–material interface is growing. The ability to substantially increase the number of screened positions without a significant cost increase for experimentation has led to increased interest in and use of gradient material samples in biological studies. As use of gradient samples in biological studies of cell–material interface continue to increase, so will the speed of our understanding of the complexity of these interactions. Material samples containing gradients in composition, material and mechanical properties, and bioactive signaling are powerful tools that have identified threshold condition in matrixes that alter cellular pathway stimulation and cellular behavior that have not observed to the same degree with tradition or other high-throughput approaches. Understanding these transition zones will enable tighter design and control of tissue formation or cytokine production in matrices. This will move tissue engineering and high-throughput screening platforms of tissue mimics closer to clinical application and potentially lead to another quantum leap forward in our understanding of cellular biology.
